# Dataset of blood cockle (*Anadara granosa*) microbiota from coastal areas and earthen-pond farms around the upper Gulf of Thailand

**DOI:** 10.1016/j.dib.2020.105393

**Published:** 2020-03-10

**Authors:** Monnat Theerachat, Chompunuch Glinwong, Warawut Chulalaksananukul

**Affiliations:** a Department of Botany, Faculty of Science, Chulalongkorn University, Bangkok 10330, Thailand; b Biofuels by Biocatalysts Research Unit, Faculty of Science, Chulalongkorn University, Bangkok 10330, Thailand

**Keywords:** Blood cockle, Bivalve, Mollusc, *Anadara granosa*, Metagenomics, 16S rRNA gene, Heavy metals

## Abstract

The blood cockle (*Anadara granosa*), a bivalve mollusc, is a unique seafood item in Southeast Asia. Bivalve molluscs are filter feeders upon plankton, and so they may bioaccumulate microbes and heavy metals in their tissues. Bacteria survival can be enhanced by living inside the shell and they can subsequently infect humans and higher vertebrates after ingestion of the bivalve. This study presented a metagenomics analysis of the bacteria associated with *A. granosa* from six farms around the Gulf of Thailand. Three farms were located on the coast and the other three were from earthen ponds. Genomic DNA was extracted from the samples and analysed via sequencing of the V3–V4 region of the 16S rRNA gene, and then using a 97% DNA sequence similarity cut-off for designation of the operational taxonomic units. The environmental parameters, including temperature, pH, salinity, dissolved oxygen, and the concentration of three heavy metals (Cu, Cr, and Hg) and one metalloid (As) were investigated. The raw sequence data is available at the NCBI Sequence Read Archive accession number PRJNA592226. The Proteobacteria, Bacteroidetes and Cyanobacteria were common components of the microbiota in all six habitats and together comprised more than 77% of the relative abundance in all the samples. This is the first report on the microbiome in blood cockles in Thailand by a culture independent method. The data can be applied for efficiently controlling and improving seafood safety management.

Specifications table

**Table:** 

Subject	Biology
Specific subject area	16S rRNA sequencing
Type of data	Table Figure Raw V3–V4 16S rRNA gene sequence reads
How data were acquired	Sequences were acquired by Illumina MiSeq. Water temperature, salinity and dissolved oxygen were recorded at the sampling site with a YSI 556 Multiprobe system. Concentration of three heavy metals and one metalloid in blood cockles was determined in each sample by atomic absorption spectroscopy (AAS).
Data format	Raw and analysed data
Parameters for data collection	Blood cockles were collected in December 2016 from six sampling sites around the upper Gulf of Thailand (GOT), where the culture of blood cockles is an activity of great socio-economic importance. Three sampling sites were located on the coast, the other three were at earthen ponds located slightly inland. These two types of locations were chosen so as to compare the location factor that may shape the bacterial community.
Description of data collection	Blood cockles were collected and stored on ice during the transportation to the laboratory. In the laboratory, each blood cockle was carefully shucked using sterile knives and subjected to DNA extraction from 250 mg of tissue within 24 h of collection. The 16S rRNA gene libraries were constructed using V3–V4 primers and sequenced on an Illumina MiSeq.
Data source location	Data was analysed at Chulalongkorn University, Bangkok, Thailand Sampling locations: CHSE: 13°21′47.14″ N, 100°58′14.64″ E PHSE: 13°15′53.94″ N, 99°57′6.18″ E CCSE: 13°17′58.32″ N, 99°58′38.72″ E CCSO: 13°20′23.94″ N, 99°58′38.31″ E BPSO1: 13°29′14.52″ N, 100°15′32.03″ E BPSO2: 13°30′0.47″ N, 100°15′26.12″ E
Data accessibility	Data is available with this publication. The raw sequence reads of all samples have been deposited at the NCBI Sequence Read Archive (SRA) under accession number PRJNA592226 (https://www.ncbi.nlm.nih.gov/sra/PRJNA592226).

## Value of the data

• This project represents the diversity of bacteria communities of *A. granosa*, cultured in six farms located on the coast and earthen ponds around the upper GOT, by 16S rRNA gene sequencing.
• Microbial community data of *A. granosa* can act as a reference for other researchers interested in bivalve mollusc-associated bacteria and food safety.
• The data can be applied for the efficient control and improvement of seafood safety management and to gain a better understanding of the bacterial community in *A. granosa*.
• Results on the heavy metal levels in *A. granosa* and the environmental parameters can used as a baseline for the present state of heavy metals in *A. granosa* in the GOT.

## 1 Data

The blood cockle (*Anadara granosa*), a bivalve mollusc, is a source of cheap protein that is widespread in Thailand. Blood cockle farms in the upper GOT are one of the main sources of blood cockle trade, and retail for 180–200 THB (∼6–7 $)/kg with about 60 cockles/kg. Currently, the Thai coasts on the GOT and Andaman sea are comprised of large barrier beaches and mud flats. Practically, shellfish in the ocean can be a sound habitat for a variety of marine bacteria. Bivalve molluscs are filter feeders upon plankton, and so they can bioaccumulate viruses, bacteria, and heavy metals in their tissues. Bacteria survival can be enhanced by living inside the bivalve's shell and they can subsequently infect humans and higher vertebrates upon ingestion of the bacteria [1,2]. The bacterial communities associated with the tissues of bivalves such as haemolymph and digestive gland are dominated by members of the *Vibrio* and *Pseudoalteromonas* genera [3].

Only a small portion of the bacteria in nature can be grown clonally in the laboratory using standard culture techniques [4], which restricts identification and quantification of bacterial communities. However, DNA sequencing technologies have provided new perspectives in studying microbial communities associated with animal and human tissues. These microbiotas play many important roles in animal and human health, such as nutrient processing and protection from diseases [5]. Thus, the objective of this project was to survey the bacterial community in blood cockles by using 16S rRNA gene sequencing as a basis to potentially reduce problems in bivalve aquaculture, in parallel with the environmental parameter investigation, including temperature, salinity and dissolved oxygen. The study area was comprised of six sites located in the upper GOT, three coastal sites and three slightly inland earthen ponds. The data can be applied for the efficient control and improvement of seafood safety management in Thailand.

**Fig. 1:**
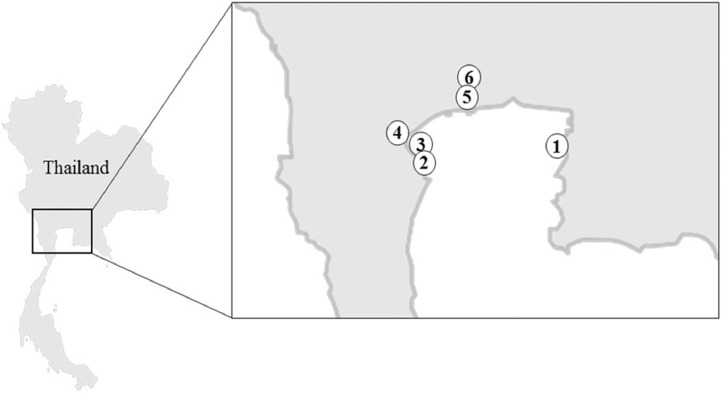
Location of the six sampling sites: (1) CHSE, (2) PHSE, (3) CCSE, (4) CCSO, (5) BPSO1, and (6) BPSO2. The CHSE, PHSE, and CCSE farms are located on the coast, while the CCSO, BPSO1, and BPSO2 farms are inland earthen pond farms. Location and farm codes are given in Table 1.

**Fig. 2:**
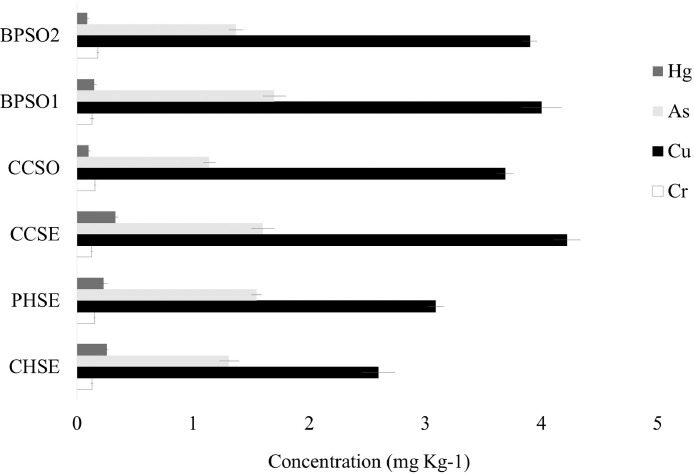
Heavy metal/metalloid concentration in blood cockles at each of the six locations. Data are shown as the mean ± 1SD of three replicates. Location (farm) codes are given in Table 1 and Fig. 1.

**Fig. 3:**
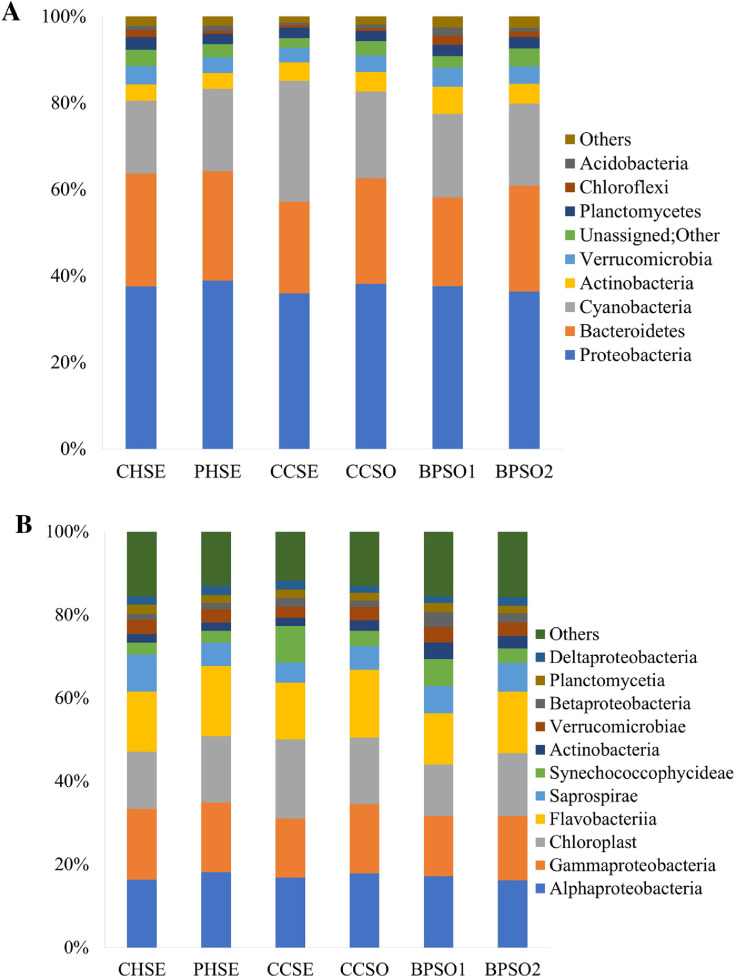

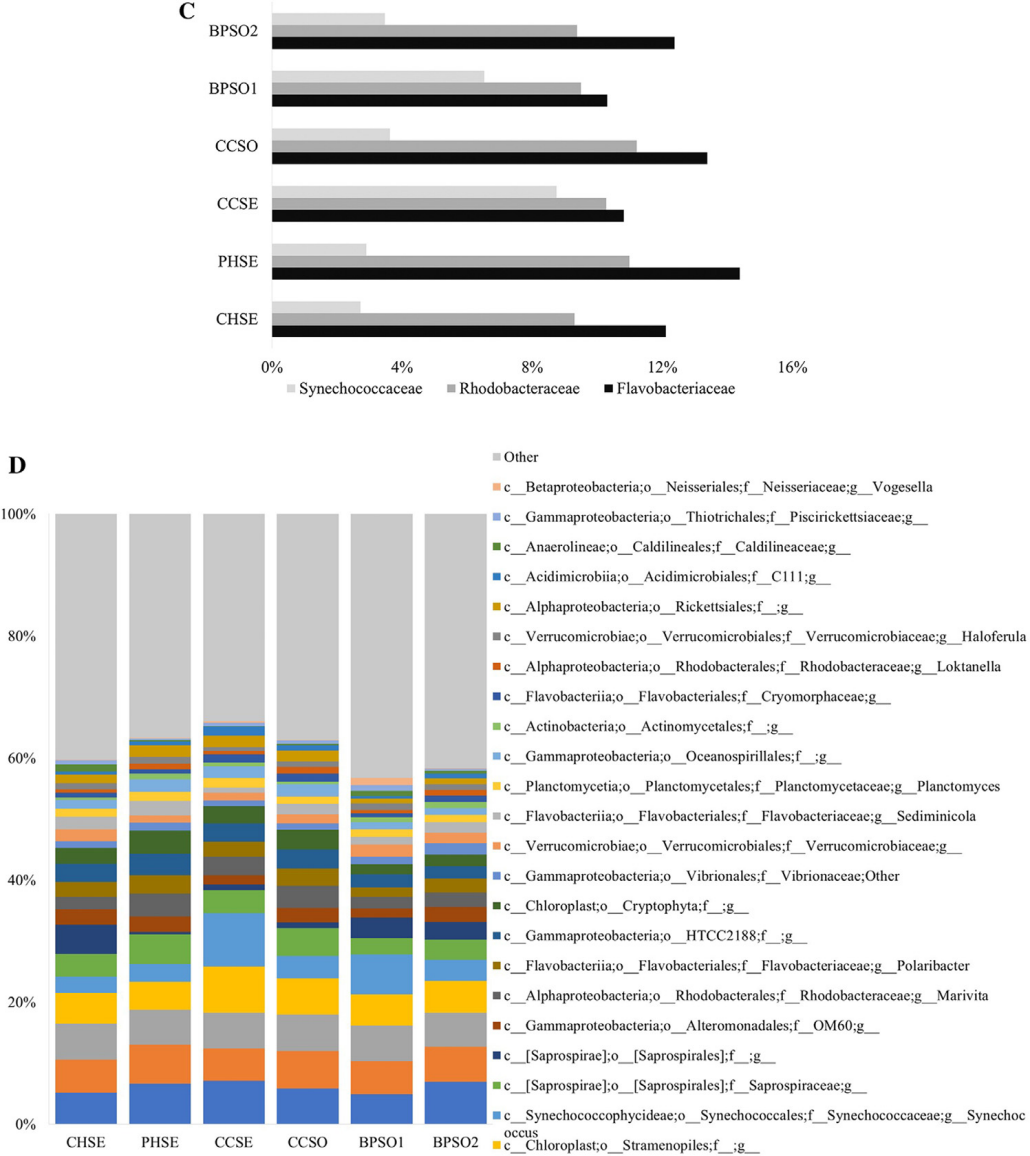
Relative microbial abundances in blood cockles at the six studies sites based on 16S rRNA gene sequences at the (A) phylum and (B) class level. Classifications with less than 1% abundance are arranged into “other”. (C) Relative abundance (%) of the top three families. (D) Relative abundance (%) at the genus level. Classifications with less than 1% abundance are arranged into “other”. Location and farm codes are given in Table 1 and Fig. 1.

**Fig. 4:**
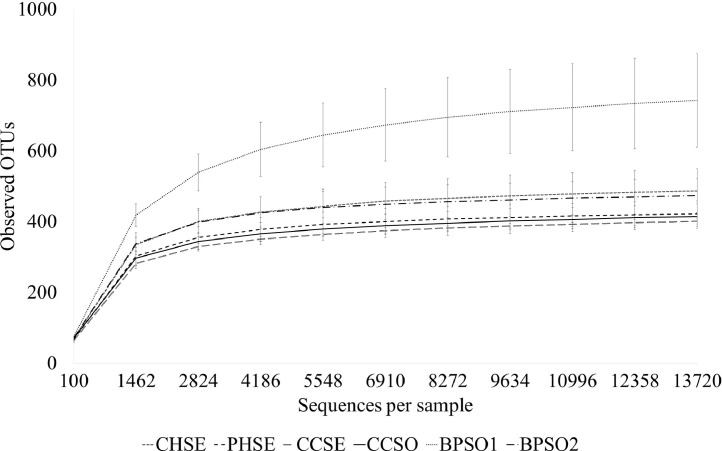
Rarefaction curves for the blood cockles from the inland earthen pond (CCSO, BPSO1, and BPSO2) and coastal (CHSE, PHSE, and CCSE) farms showing the number of OTUs and the number of sequences analyzed. Location and farm codes are given in Table 1 and Fig. 1.

**Fig. 5:**
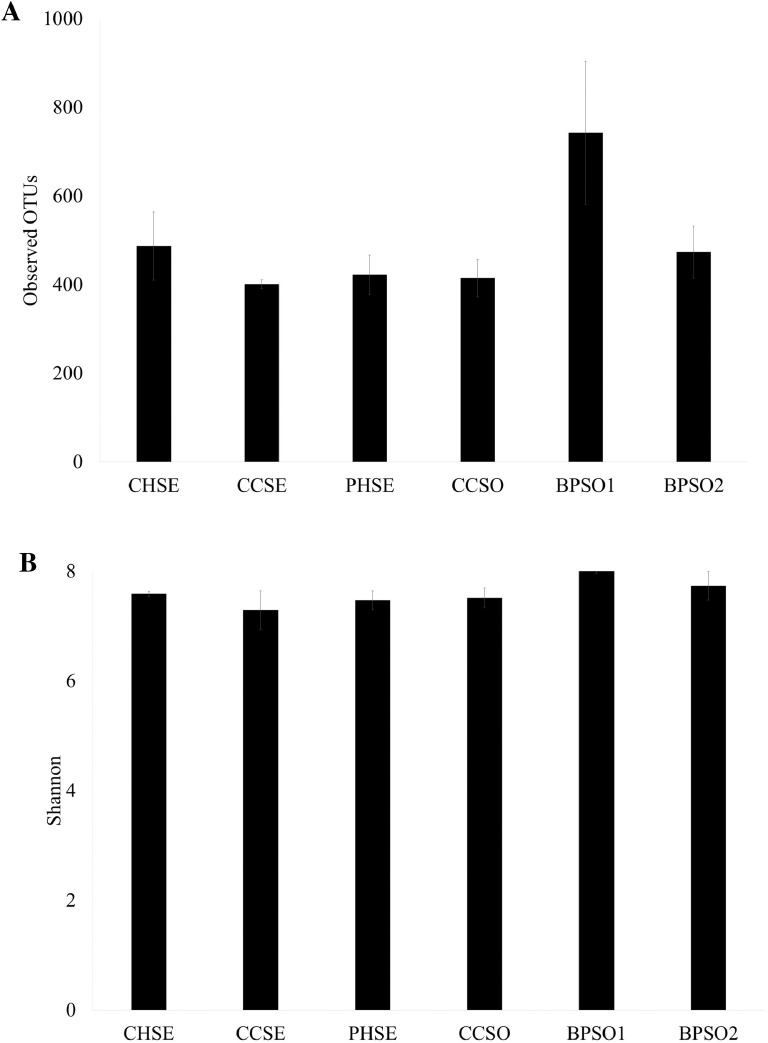

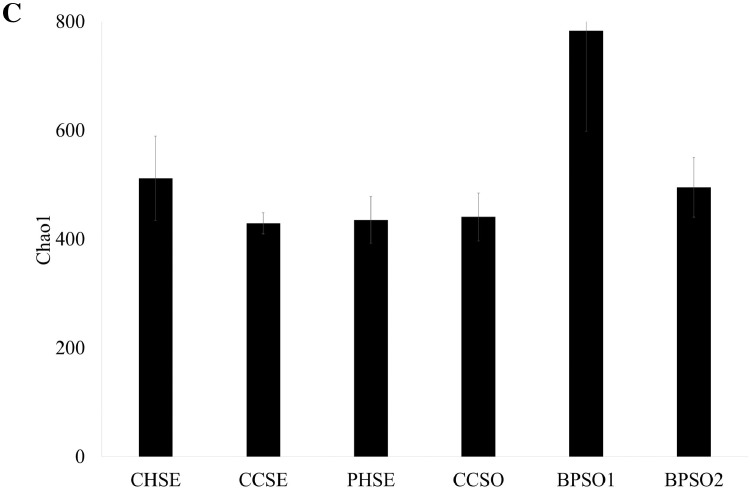
Biodiversity indexes, shown as the (A) observed OTUs, (B) Shannon-Wiener index, and (C) Chao1 index. Data are shown as the mean ± 1 SD (error bar), derived from three independent replicates. Location and farm codes are given in Table 1 and Fig. 1.

**Fig. 6:**
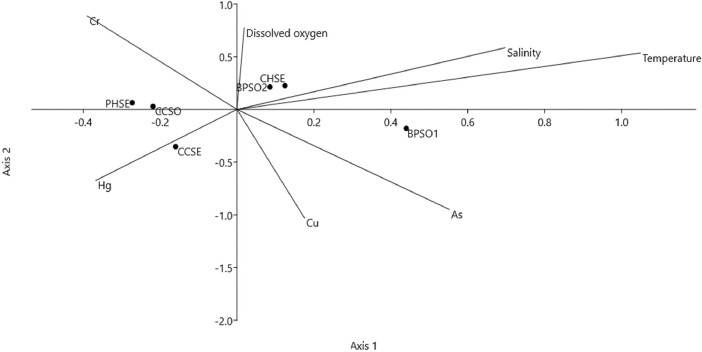
The CCA of heavy metal/metalloid content, dissolved oxygen content, salinity, and temperature on the biodiversity indexes for the coastal (CHSE, PHSE, and CCSE) and earthen pond (CCSO, BPSO1, and BPSO2) blood cockle farms. Location and farm codes are given in Table 1 and Fig. 1.

The dataset contains raw sequencing data obtained by 16S rRNA sequencing of blood cockles from six habitats. The sequence data were deposited at the NCBI Sequence Read Archive (SRA) under accession number PRJNA592226. Sampling sites and the concentration of selected heavy metals/metalloid in the blood cockles are shown in Figs. 1 and 2, respectively. Information about the location, physical and chemical water quality measurements, including the temperature, salinity, and dissolved oxygen content, at the sampling sites are presented in Table 1. The relative microbial abundances at the phylum, class, family and genus level in the blood cockles based on 16S rRNA gene sequences are revealed in Fig. 3, while Figs. 4 and 5 show the rarefaction curves and biodiversity indexes, respectively. The canonical correspondence analysis (CCA) of the heavy metal/metalloid contents in *A. granosa* and environmental parameters, including dissolved oxygen, salinity, and temperature, on the biodiversity indexes for the six farms are shown in Fig. 6.

**Table 1:** Location, physical and chemical water quality measurements, including temperature, salinity, and dissolved oxygen content, at the sampling sites.

Sample name	Area	Latitude/longitude	Temperature (°C)	Salinity (ppt)	Dissolved oxygen (mg/L)
CHSE	Coast	13°21′47.14″ N, 100°58′14.64″ E	29.4 ± 0.1	28.5 ± 0.2	4.5 ± 0.1
PHSE	Coast	13°15′53.94″ N, 99°57′6.18″ E	26.5 ± 0.1	10.8 ± 0.1	1.1 ± 0.0
CCSE	Coast	13°17′58.32″ N, 99°58′38.72″ E	27.5 ± 0.1	10.3 ± 0.0	2.0 ± 0.0
CCSO	Earthen pond	13°20′23.94″ N, 99°58′38.31″ E	28.5 ± 0.0	10.5 ± 0.1	3.5 ± 0.0
BPSO1	Earthen pond	13°29′14.52″ N, 100°15′32.03″ E	28.9 ± 0.1	15.8 ± 0.1	1.4 ± 0.1
BPSO2	Earthen pond	13°30′0.47″ N, 100°15′26.12″ E	28.7 ± 0.1	10.5 ± 0.1	2.6 ± 0.0

## 2 Experimental design, materials and methods

### 2.1 Sampling, heavy metals analysis

Blood cockles (*Anadara granosa*) were collected in December 2016 from six sampling sites around the upper GOT, where the culture of blood cockles is an activity of great socio-economic importance. Three sampling sites were coastal, while the other three were slightly inland earthen ponds. Blood cockles were stored on ice during transportation to laboratory. The water temperature (°C), salinity (ppt), and dissolved oxygen content (mg/L) were recorded at the time of sampling at each site using a YSI 556 Multiprobe system (YSI Incorporated, USA) in accordance with the manufacturer's instruction. In the laboratory, each blood cockle was cleaned with sterile distilled water to remove sand and mud, and carefully shucked using sterile knifes. The blood cockles were then used for DNA extraction within 24 h of collection. The concentration of three heavy metals (Cr, Cu, and Hg) and one metalloid (As) was determined in each sample by AAS.

### 2.2 Evaluation of bacterial communities

#### 2.2.1 DNA extraction and sequencing

For genomic DNA extraction, 200 g of blood cockles were homogenized and then DNA extraction was performed using a DNA purification kit (NucleoSpin^Ⓡ^Soil, Macherey-Nagel) according to the manufacturer's instruction. Sterile distilled water was used for the final elution. The DNA was then stored at −20 °C until analysis.

#### 2.2.2 Bioinformatic analysis

The V3–V4 16S rRNA region primers were used for construction of the 16S rRNA gene libraries. Amplicons were achieved using a high-fidelity polymerase and 2X KAPA hot-start ready mix. Thermal cycling was performed at 94 °C for 3 min, then 25 cycles of 98 °C for 20 s, 55 °C for 30 s, and 72 °C for 30 s, and then followed by a final 72 °C for 5 min. The amplicons were then purified and indexed using 2X KAPA hot-start ready mix and 5 µL of each Nextera XT index primer with 8–10 cycles of the same PCR condition as described above. The AMPure XP beads were used to purify the products. After that, they were pooled and diluted to a final loading concentration of 6 pM. It should be noted that the products were pooled to increase the number of sequenced samples per run. Each sequenced sample can be identified and separated from the others later using Nextera XT index primer sequences attached individually as describe above. Cluster generation and 250-bp paired-end read mode of sequencing were performed on an Illumina MiSeq at Omics Sciences and Bioinformatics Center (Chulalongkorn University, Bangkok, Thailand). The FASTQC and PEAR software were used for quality control of raw sequencing reads and assembly, respectively.

Raw sequences were filtered using the FASTX-Toolkit with a threshold quality score of 30. Reads that were shorter than 400-bp were also filtered. Chimeras were eliminated using the UCHIME method [6]. Selection of OTU picking was obtained using the pick_open_reference_otus.py command in QIIME 1.9.0 [7], while SortMeRNA was used for reference picking. Greengenes database were then used to compare for taxonomic assignments to convert the OTUs to likely species using a 97% sequence similarity cutoff. The subsampled failure reads were then clustered de novo using SUMACLUST. Those OTUs that were supported by less than 0.1% reads were eliminated. The resulting 13,729 sequences obtained were subsampled for bacterial community analysis and a rarefaction curve was generated.

#### 2.2.3 Statistical analysis

To study the influence of environmental parameters (concentration of Cr, Cu, Hg, and As, plus the temperature, salinity, and dissolved oxygen content) on the microbial communities between different sampling sites, CCA with 999 permutations using PAST3 was performed.

## Appendix Supplementary materials

Image, application 1

Image, application 2

Image, application 3
